# Implementation of STOMP (Stopping Over-Medication of People With Learning Disability, Autism or Both With Psychotropic Medications) PLEDGE: A Quality Improvement Project at Bradford District Care Foundation Trust CAMHS Learning Disability Team

**DOI:** 10.1192/bjo.2022.343

**Published:** 2022-06-20

**Authors:** Mahira Syed, Sarojit Ganguly

**Affiliations:** Bradford District Care NHS Foundation Trust, Bradford, United Kingdom

## Abstract

**Aims:**

The project's aim coincides with NHS England STOMP Pledge signed by BDCFT. To maintain up to date records of children and adolescents with learning disabilities eligible for STOMP reviews, implement planned supervised dose reduction, consider alternatives to psychotropics and maintain an up-to-date record of physical health monitoring for patients on antipsychotic medications according to local Trust guidelines.

**Methods:**

The sample consisted of the caseload registered with the CAMHS learning disability Team at BDCFT in December 2021. Each case was reviewed retrospectively through electronic records. Data were collected on a data collection tool designed in Microsoft Excel.

Baseline data about Diagnosis and Psychotropic medications prescribed were recorded. The Antipsychotic prescribing practice was audited against local Trust guidelines as part of the project.

The project was registered and approved by the Trust Audit Team.

**Results:**

The study included 106 cases registered in December 2021.

42 patients (40%) were prescribed psychotropic medication only

10 patients (9%) were prescribed psychotropic medication plus ADHD medication

14 patients (13%) were prescribed ADHD medication only

40 patients (38%) were not prescribed any medication

66 (62%) patients from the sample were prescribed medication.

Medications were divided into, Psychotropics and ADHD medication groups. Each group was assessed against a prescription time standard of either less or more than 12 months.

Antipsychotics were the most frequently prescribed psychotropic medications; 60% of those prescribed psychotropics were on Antipsychotics. A smaller number (31%) on an Anxiolytic, and an even small number (12%) on an Antidepressant. Anticonvulsants were prescribed to 6 in our sample, but all by another service provider (Paediatrics). 20 patients (38%) were on more than one psychotropic medication.

The length of the time was divided into less and more than a year on medication. 20% of patients were on psychotropics for less than 12 months and about 80% for more than 12 months.

As there are local BDCFT guidelines for monitoring patients on Antipsychotics, a summary of compliance against standards was included as an audit in the project.

All 66 patients on medications were deemed eligible for STOMP reviews, and 64 out of them had behavioural support plan in place.

**Conclusion:**

66 patients who had eligibility for STOMP:

1. 35%: Undergoing reduction plan.
2. 35%: Reduction was not deemed suitable.
3. 30%: No review or reduction plan in place

Recommendations are made in the report to achieve full compliance with STOMP objectives and a re-audit in a year to monitor progress.

